# Impact of Graded Passive Cycling on Hemodynamics, Cerebral Blood Flow, and Cardiac Function in Septic ICU Patients

**DOI:** 10.3389/fmed.2020.569679

**Published:** 2020-10-16

**Authors:** Jennifer Chen, Claudio Martin, Ian M. Ball, Christopher W. McIntyre, Marat Slessarev

**Affiliations:** ^1^Department of Medical Biophysics, Western University, London, ON, Canada; ^2^Department of Medicine, Western University, London, ON, Canada; ^3^Departments of Epidemiology and Biostatistics, Western University, London, ON, Canada; ^4^The Brain and Mind Institute, Western University, London, ON, Canada

**Keywords:** passive cycling, passive exercise, hemodynamics, cerebral blood flow, cardiac contractility, sepsis, critical care

## Abstract

**Background:** In-bed passive cycling is considered a safe and feasible early mobilization technique in intensive care unit (ICU) patients who are unable to exercise actively. However, the impact of varying intensity of passive cycling on perfusion and function of ischemia-prone organs is unknown. In this study, we assessed the impact of a graded passive cycling protocol on hemodynamics, cerebral blood flow, and cardiac function in a cohort of septic ICU patients.

**Methods:** In consecutive patients presenting with sepsis, we measured global hemodynamic indices, middle cerebral artery velocity (MCAv), and cardiac function in response to a graded increase in passive cycling cadence. Using 5-min stages, we increased cadence from 5 to 55 RPM in increments of 10 RPM, preceded and followed by 5 min baseline and recovery periods at 0 RPM. The mean values obtained during the last 2 min of each stage were compared within and between subjects for all metrics using repeated-measures ANOVA.

**Results:** Ten septic patients (six males) completed the protocol. Across patients, there was a 5.2% reduction in MCAv from baseline at cycling cadences of 25–45 RPM with a dose-dependent decrease of MCAv of > 10% in four of the 10 patients enrolled. There was a 16% increase in total peripheral resistance from baseline at peak cadence of 55 RPMs and no changes in any other measured hemodynamic parameters. Patient responses to passive cycling varied between patients in terms of magnitude, direction of change, and the cycling cadence at which these changes occurred.

**Conclusions:** In septic patients, graded passive cycling is associated with dose-dependent decreases in cerebral blood flow, increases in total peripheral resistance, and either improvement or worsening of left ventricular function. The magnitude and cadence threshold of these responses vary between patients. Future studies should establish whether these changes are associated with clinical outcomes, including cognitive impairment, vasopressor use, and functional outcomes.

## Introduction

Sepsis is associated with short- and long-term complications, including physical and cognitive impairment (1, 2). Although early active mobilization is associated with improvements in peripheral strength at hospital discharge (3, 4) and shorter delirium duration (5), many patients are unable to participate in active mobilization during earlier stages of critical illness due to decreased levels of consciousness and ventilator dependence. In these patients, passive exercise has been reported as a safe and feasible method to incorporate mobilization early in the course of their ICU stay (6), and it is also associated with improved patient motivation during recovery following critical illness (7). However, prior studies that assess the safety and feasibility of early mobilization protocols in critically ill patients primarily do so by confirming the stability of global hemodynamic variables and minimal occurrences of adverse events (3, 8). The impact of passive exercise on perfusion of ischemia-prone organs, such as the brain and the heart, remain unknown in critically ill patients.

In this study, we assessed the impact of graded passive cycling on global hemodynamics, cerebral blood flow, and cardiac function in a cohort of septic patients. Given that early sepsis is associated with hemodynamic instability (9), tenuous organ perfusion (10), and impaired cerebral autoregulation (11), it is important to confirm that graded passive cycling does not result in impaired perfusion and function of ischemia prone organs prior to wide implementation of this promising intervention in critically ill patients.

## Materials and Methods

### Subjects

Following Institutional Research Ethics Board approval, we obtained informed written consent from patients or their surrogate decision maker and prospectively enrolled 10 consecutive adult (age 18 and older) septic patients admitted to our medical-surgical ICU. Sepsis was defined based on the Sepsis-3 definition (12). We recorded participants' age, weight, and height as well as resting blood pressure at the start and the end of the study protocol. We employed the same passive exercise protocol and similar methodology as our prior study conducted in healthy adults (13).

### Global Hemodynamic Monitoring

We used Finapres® NOVA (Finapres Medical Systems, Amsterdam, Netherlands) to measure beat-by-beat arterial blood pressure, heart rate, stroke volume, cardiac output, and total peripheral resistance using pulse wave analysis. The appropriate cuff was sized for each subject and applied to the middle finger, and the height correction unit was placed at the level of the heart. We used participants' height and weight to calculate their body surface area, which we used to compute indices of stroke volume (SVI), cardiac output (CI), and total peripheral resistance (TPRI) obtained from Finapres. Additional monitoring of mean arterial pressure and heart rate were conducted through routine care intra-arterial hemodynamic monitoring.

### Cerebral Blood Flow Monitoring

We used transcranial Doppler (TCD, Spencer Technologies, Redmond, USA) to measure the mean velocity of blood flow in the middle cerebral arteries (MCAv) as a marker of global cerebral blood flow (CBF). Although advanced tomographic techniques, such as computed tomography or magnetic resonance imaging, offer more accurate assessments of CBF, their use would require patients to be transported from the ICU to the imaging department. Given that we aimed to study patients early in the course of their critical illness, transcranial Doppler provided a safer alternative for monitoring exercise-induced changes in CBF with high temporal resolution, and it is an accepted practice for monitoring cerebral hemodynamics in critical care (14). After adequate signals were attained using standard techniques (15), Doppler probes were fixed in place using a head harness (Spencer Technologies, Redmond, USA) to ensure the same angle of insonation and adequate signal power. Data was recorded continuously at 125 Hz using provided software.

### Cardiac Function Monitoring

We used standard gray-scale 2-D echocardiography (Vivid I® with 1.5–3.6 MHz imaging transducer, GE Medical Systems, Sonigen, Germany) to collect apical 2 and 4-chamber views of the left ventricle (LV) at each experimental stage. Images were collected with a frame rate of 70–90/s by an experienced and trained echocardiographer and saved digitally for off-line analysis. We used speckle tracking software (Echo-PAC Dimension, GE Healthcare, Germany) to determine ejection fraction as well as global longitudinal strain (GLS) as previously described (16). After tracing the endocardial border in both views of the LV at end systole, the software automatically selected stable acoustic objects within the myocardium to track and compute strain throughout the cardiac cycle. The LV was divided into 6 segments per view (12 in total) with the peak systolic strain values calculated for each segment (segmental strain) and GLS calculated as the mean of all 12 segments. Segments that failed to track were manually adjudicated. Previous studies have demonstrated that healthy individuals have GLS ranging from −16 to −19% [less negative values correspond to reduced contractility (17)].

### Passive Cycling Protocol

**Figure 1:**
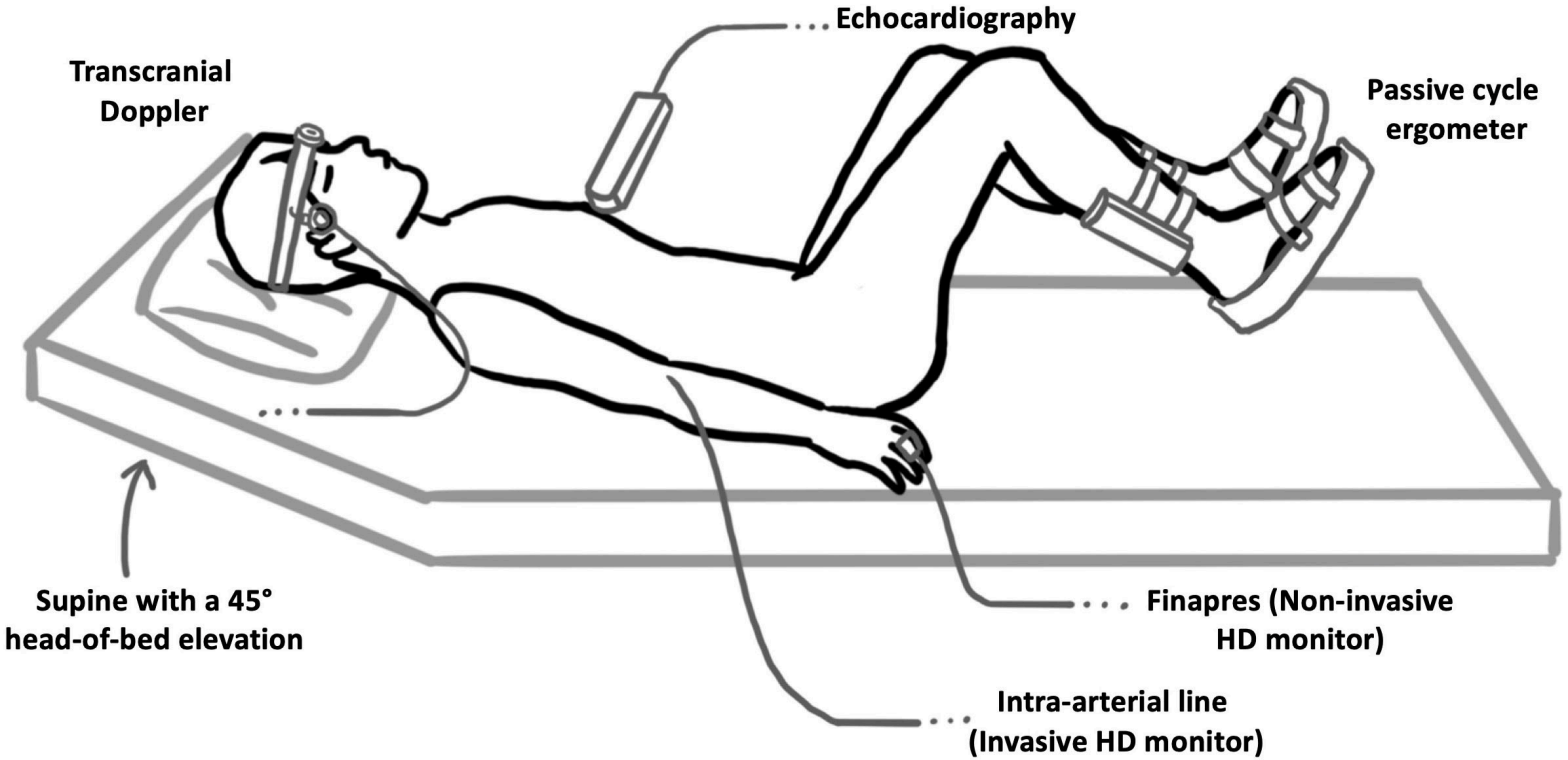
Patient and experimental setup used. Patients were positioned supine with a 45° head-of-bed elevation and passively cycled using an in-bed cycle ergometer. Transcranial Doppler was used to measure the velocity of blood flow in the middle cerebral arteries (as a surrogate marker of global cerebral blood flow). Gray-scale 2-D echocardiography was used to collect apical 2- and 4-chamber views of the LV at each cycling intensity, which were then used to compute patient EF and GLS at each experimental stage. We used Finapres® NOVA to measure hemodynamic parameters using pulse wave analysis with additional mean arterial pressure and heart rate monitoring performed through routine care intra-arterial hemodynamic monitoring.

Using the same passive cycling protocol as our prior study in healthy participants (13), passive in-bed cycling was delivered to septic patients using a clinical in-bed cycling ergometer (RT300 Supine Cycle, Restorative Therapies, Baltimore, USA) that allowed both setting and measurement of cadence to ensure that the subject was not applying any force to the ergometer (i.e., exercise intervention remaining passive). Patients were studied in the supine position with 45° head-of-bed elevation. After positioning patients in bed, we secured their legs to the bike using the straps provided as per standard protocol. We ensured that the bike and leg positioning was optimal to enable full passive leg range of motion. An illustration of the patient and equipment setup is included in Figure 1.

**Figure 2:**
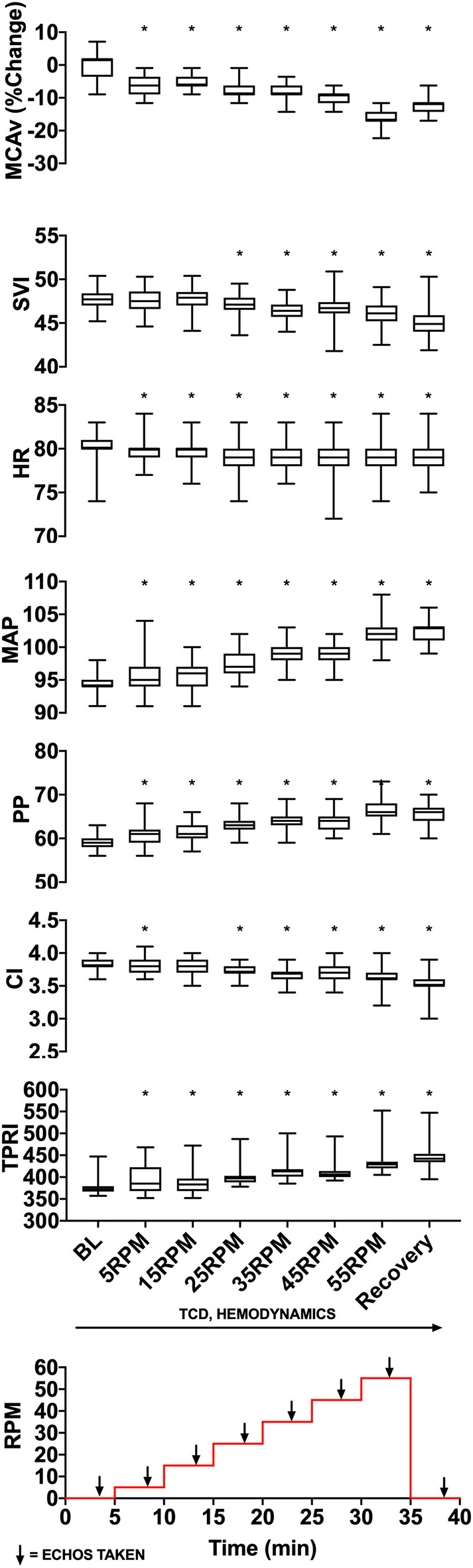
Experimental data from a representative patient showing changes in MCAv (percentage change from baseline, cm/s), SVI (mL/m^2^/beat), HR (beats per minute), MAP (mmHg), PP (mmHg), CI (L/min/m^2^), and TPRI (mmHg /m^2^ L min) with increasing cycling cadence. The bottom panel illustrates the experimental protocol with eight 5-min stages starting from 0 RPM baseline, then increasing from 5 to 55 RPMs in increments of 10 RPM and followed by a 0-RPM recovery stage. Monitoring of cerebral blood flow using transcranial Doppler and hemodynamics occurred continuously, and cardiac function was assessed using transthoracic 2-D echocardiography during the last 2 min of each stage. The median, lower quartile, upper quartile, and the min and max are illustrated describing values from a single patient. MCAv values are standardized and expressed as the percentage change from subject's resting baseline value because the angle of insonation may affect absolute MCAv and these angles can vary between subjects. *p < 0.05 as measured with repeated-measures ANOVA.

We used TCD probes to identify MCAv in one or both middle cerebral arteries. We applied the Finapres probe to the middle finger of patient's right hand and ensured that we could measure beat-by-beat blood pressure and other hemodynamic parameters. Following patient acclimatization to the equipment (~10 min), we started the experimental protocol (Figure 2). The protocol consisted of 8 stages, each lasting 5 min. Starting at baseline (0 RPM), the cadence on the bicycle was increased in stages from 5 to 55 RPM in increments of 10 RPM, followed by a 5-min recovery period at 0 RPM. TCD and Finapres data were acquired continuously, and echocardiography was performed during the last 2 min at each cadence stage and baseline/recovery periods.

### Data Analysis

**Figure 3:**
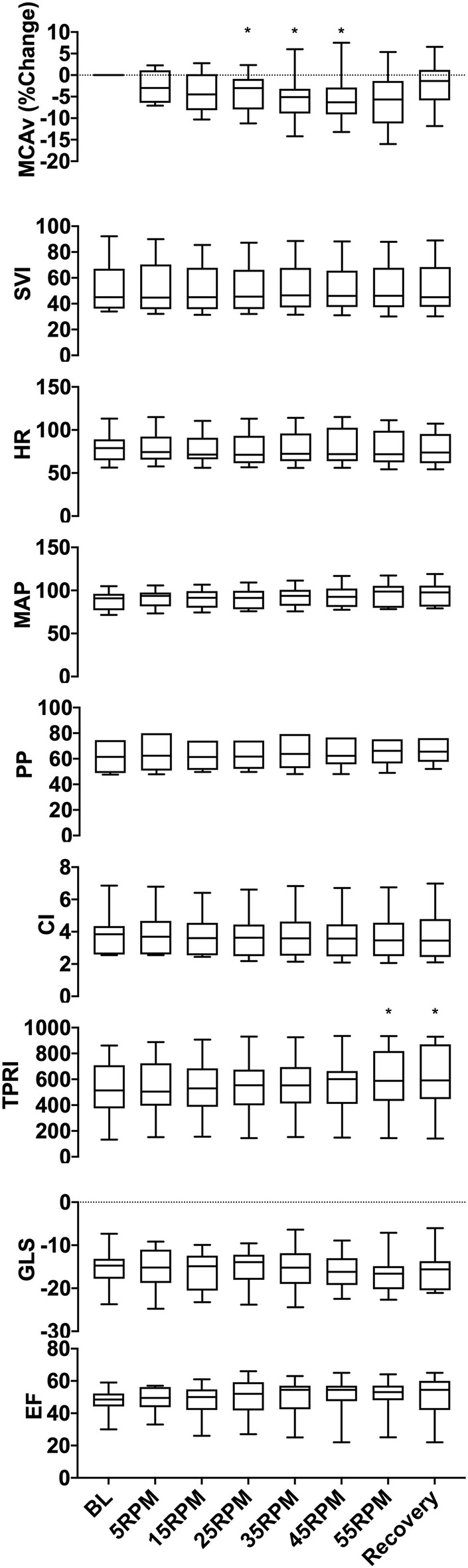
Group data showing changes in MCAv, SVI, HR, MAP, PP, CI, TPRI, GLS, and EF with increasing cycling cadence. The box plots represent values averaged at each passive cycling stage across all patients. A significant decrease was seen in MCAv between 25 and 45 RPM (*p* = 0.004), and an increase in TPRI was observed at 55 RPM (*p* = 0.008), which persisted throughout the recovery period. No significant changes were seen in the remaining hemodynamic parameters (SVI, HR, MAP, PP, CI, and TPRI) or in either assessment of cardiac function (GLS and EF). *p < 0.05 as measured with repeated-measures ANOVA.

A total of 11 parameters were analyzed: MCAv, mean arterial pressure (MAP), systolic blood pressure (SPB), diastolic blood pressure (DPB), pulse pressure (PP), heart rate (HR), CI, SVI, and TPRI, left ventricular ejection fraction (EF), and left ventricular GLS. For each patient, data collected by the TCD and Finapres were exported to Excel (Microsoft) and sorted by experimental stage (baseline 0 RPM, 5 RPM, 15 RPM, 25 RPM, 35 RPM, 45 RPM, 55 RPM, or recovery 0 RPM). Data from the last 2 min of each stage were used to calculate mean and standard deviation for that stage. MCAv values were expressed as the percentage change from each patient's baseline value in order to allow between-patient comparisons (absolute MCAv values depend on the angle of insonation, which can differ between subjects). Echocardiographic data (EF and GLS) from each stage was also recorded. These metrics were exported to statistical software (Prism 7, GraphPad, San Diego, USA) that was used to construct graphs of measured parameters vs. experimental stage for individual patients (Figure 2) and for the whole group (Figure 3). We inspected the graphs visually for trends. We used descriptive statistics to report demographic data and repeated-measures one-way ANOVA to assess differences in metrics within patients and group differences between patients. Statistical significance was assumed when *p* < 0.05.

## Results

### Patient Demographics

Ten septic patients (4 females and 6 males) were enrolled, and all patients successfully completed the study protocol. The median (IQR) age, body mass (kg), and sequential organ failure assessment (SOFA) score were 56.5 (7.8), 82 (31), and 7.5 (3.4), respectively. At the time of enrollment, all patients were intubated, 6 (60%) were treated with vasopressors, and 8 (80%) received sedation with propofol, hydromorphone, fentanyl, or a combination of these three sedatives. The median (IQR) duration from patients' ICU admission to participation in passive cycling was 2 days. Patient baseline characteristics are summarized in Table 1.

**Table 1:** This table summarizes patient demographics and patient outcomes.

**Characteristic**	***n* = 10 Patients**
Age in years, median (IQR)	56.5 (7.8)
Males, *n* (%)	6 (60)
Height in cm, median (IQR)	170 (11)
Body mass in kg, median (IQR)	82 (31)
Comorbidities, *n* (%)	
Hypertension	4 (40)
Congestive heart failure	2 (20)
Atrial fibrillation	2 (20)
Pneumonia	1 (10)
COPD	1 (10)
Stroke	1 (10)
SOFA score, median (IQR)	7.5 (3.4)
Vasopressors, *n* (%)	6 (60)
Levophed	6 (60)
Vasopressin	3 (30)
Phenylephrine	1 (10)
Amiodarone	1 (10)
Sedatives, *n* (%)	8 (80)
Propofol	8 (80)
Hydromorphone	7 (70)
Fentanyl	1 (10)
Duration from ICU admission to conduction of passive cycling protocol in days, median (IQR)	2 (2)
ICU LOS in days, median (IQR)	16 (8)
ICU mortality, *n* (%)	4 (40)
Hospital LOS in days, median (IQR)	22 (14)
Hospital mortality, *n* (%)	4 (40)
Duration of MV in days, median (IQR)	10 (11)

*IQR, interquartile range; COPD, chronic obstructive pulmonary disease; SOFA, Sequential Organ Failure Assessment, a 6-item instrument with scores from 0 to 24 with higher scores indicating more severe organ failure, ICU, intensive care unit; LOS, length of stay; MV, mechanical ventilation*.

### Hemodynamics

Figure 2 illustrates the experimental protocol used, which was previously tested for safety and feasibility in a healthy cohort (13), along with data from a representative subject. Averaged group trends are shown in Figure 3. All data are reported as mean ± standard deviation. Across patients, increasing cadence from rest (0 RPM) to 55 RPM did not change the MAP from baseline (*p* = 0.057). There was, however, a 16% TPRI increase from baseline to 55 RPM (567 ± 264 to 658 ± 298 mmHg /m^2^·L·min, *p* = 0.008), which further increased to 18% (667 ± 308 mmHg /m^2^·L·min) during the recovery period. Passive cycling did not elicit changes in SVI, CI, HR, or PP across experimental stages and patients although there was substantial variability in the magnitude of hemodynamic responses between patients. At peak cycling intensity, three patients had a 13–19% increase in MAP, and five patients had an 11–19% increase in PP compared to baseline. All five patients who had increases in PP also demonstrated increased TPRI.

### Cerebral Blood Flow

Across patients, an increase in cadence was associated with a 5.2 ± 1.4% reduction in MCAv from baseline between 25 and 45 RPM (*p* = 0.004). The MCAv increased and approached baseline values during recovery. The magnitude of MCAv response varied across patients. In 8/10 patients, an increase in cadence resulted in a dose-dependent and statistically significant decrease (*p* < 0.05) in MCAv from baseline, and in four of these patients, MCAv decreased by 10–16% from baseline at higher cycling intensities (35–55 RPM). These reductions in MCAv occurred on the background of stable MAP. In one patient, we observed a dose-dependent increase in MCAv (7.5 ± 2.9% at 45 RPM) that was associated with a dose-dependent increase in MAP that peaked at 55 RPM (*P* < 0.0001, 78 ± 1 mmHg). In another patient, we observed a biphasic response with the initial reduction (−2.9 ± 4.0%) in MCAv at lower cycling intensities, followed by increase in MCAv (2.3 ± 5.7%) at higher intensities of ≥ 45 RPM.

### Cardiac Function

We did not observe a statistically significant change in cardiac function across patients in our cohort with respect to either EF (*p* = 0.99) or GLS (*p* = 0.99). However, upon inspection of individual patient responses, we identified two response patterns. Six patients demonstrated improvements in contractility, with lower (19–51%) GLS vs. baseline, and four patients demonstrated worsening cardiac function with increased (12–31%) GLS vs. baseline. The cadence at which these changes in GLS occurred varied across patients, ranging from 5 to 45 RPM.

## Discussion

This is the first study examining the effect of graded passive in-bed cycling on global hemodynamics, cerebral blood flow, and cardiac function in septic patients. We demonstrated that increasing passive cycling cadence is associated with a statistically significant reduction in cerebral blood flow from baseline that exceeded 10% in 4 of 10 patients in our cohort. The impact of passive cycling on cardiac function varied across patients with six patients demonstrating improvement and four patients demonstrating worsening of left ventricular contractile function as measured by GLS. These changes occurred at variable cycling cadence across patients and were not associated with changes in global hemodynamics except for dose-dependent increases in TPRI. Our data highlight the variability of individual patient responses to the same exercise stimulus and call into question the safety of a one-size-fits-all approach to passive cycling dose prescription in septic patients. Future studies should explore methods to individualize exercise prescriptions in critically ill patients that ensure patient safety while maximizing clinical benefits.

### Hemodynamics

We showed that, in septic patients, graded passive cycling is associated with an increase in peripheral resistance with no changes in other hemodynamic parameters. This contrasts previous findings from healthy participants that used the same passive cycling protocol and reported increases of MAP without changes in peripheral resistance (13). Given that sepsis is associated with peripheral vasodilation, the observed increase in peripheral resistance in our septic cohort likely represents improved vasomotor tone as a result of the passive cycling intervention. In a previous study of septic patients, 20 min of passive cycling at 30 RPM had no effect on peripheral resistance (18). The different result in our patients may reflect the longer duration (35 min) or higher cadence (10 min at RPM ≥ 45) employed in our protocol. Although, in our study, the increase in peripheral resistance was not associated with an immediate decrease in vasopressor requirements, future studies should evaluate if repeated passive cycling leads to lower vasopressor requirements and more vasopressor-free days.

### Cerebral Blood Flow

Increasing the cadence of passive cycling resulted in a dose-dependent decrease in MCAv with variable magnitudes of response across patients. These changes in MCAv were not associated with changes in MAP, suggesting that factors other than perfusion pressure may influence CBF in septic patients. These results are in contrast to our findings in healthy participants, in which passive cycling using the same protocol did not impact CBF despite a small increase in MAP at peak cadence (13). Our findings also contrast previous studies of healthy participants, in which passive exercise has been associated with an increase in MCAv (19) and regional cerebral blood flow (20, 21). Given that passive cycling is associated with the increase in leg blood flow (22, 23) and because cardiac index remained unchanged in our patients, the observed reduction in MCAv may represent vascular steal as a result of redistribution of blood flow from the brain to exercising extremities. Although we cannot directly confirm this hypothesis using our current data, as we didn't measure leg blood flow, future studies should explore this relationship by directly comparing the magnitude of changes in leg and cerebral blood flow during passive cycling in septic patients. The clinical importance of the observed reduction in MCAv in our study is unclear. However, given the high prevalence of delirium and cognitive impairment in critically ill patients (2) as well as the high burden of ischemic lesions in septic patients (24, 25), the observed reduction in MCAv is concerning and warrants further assessment and correlation with clinically relevant cognitive outcomes (1).

### Cardiac Function

The effect of graded passive cycling on cardiac function was variable across our patient cohort. Six patients showed improvement, and four patients showed worsening in left ventricular contractility as measured by the GLS. GLS is a more reproducible measure of left ventricular function (26) and offers better prognostic value than standard measurements, such as EF (27) although it can be affected by loading conditions (28), age, and sex (29). Because patients served as their own control, the observed changes in GLS are likely due to cycling-induced changes in loading conditions, myocardial blood flow, sympathetic tone, or neuroendocrine signaling. However, it is not clear why some patients had improvement and others deterioration in strain with increased cadence or whether these changes are clinically relevant. Although it is reassuring is that changes in contractile function resolved during the recovery period, the cumulative effect of these changes over the course of critical illness on clinical and functional outcomes in septic patients warrants further investigation.

### Heterogeneity of Responses

An important observation in our study is that hemodynamic, cerebral blood flow, and cardiac responses to passive cycling varied between individual patients in terms of magnitude, direction of change, and the cycling cadence at which these changes occurred. These variations emphasize the idea that, similar to regular exercise in healthy humans (30), a one-size-fits-all approach to passive cycling in critically ill patients may not be appropriate. The variable magnitude of the responses suggest that some patients may yield greater benefit (or sustain more harm) than other patients. Variable direction of the cardiac function response highlights that, despite the use of the same exercise paradigm, cardiac function may either improve or worsen in different patients. Finally, the exercise intensity (cadence) threshold for eliciting a given response differs between patients, which suggests that future clinical trials should individualize the exercise dose prescription in each patient. Although our study focused on adjusting exercise intensity, future work should assess the impact of changing exercise duration and frequency on measured parameters.

## Limitations

Due to the exploratory nature of our study, we had a small sample size, and thus, our findings should be interpreted with caution. Furthermore, because we enrolled consecutive patients presenting with sepsis, our cohort was not well-balanced with respect to sex. However, despite these limitations, all enrolled patients successfully completed the study protocol, and we demonstrated the feasibility of monitoring hemodynamic, cerebral blood flow, and cardiac function during a graded passive exercise protocol in septic ICU patients. Given that we observed a reduction in MCAv with increasing exercise cadence in 8 out of 10 patients, we decided to stop further enrollment pending full data analysis. The small sample size in our study also limits the analysis of the results with respect to important covariates, including patient age, sex, baseline health and fitness, comorbidities, and severity of illness. Future properly powered studies can explore these relationships further.

Although exercise dose has three components (intensity, duration, and frequency), our protocol was primarily focused on assessing the impact of a graded increase in exercise intensity on measured parameters. Future studies should explore the impact of exercise duration and frequency on hemodynamics, cerebral blood flow, and cardiac function in critically ill patients.

We used transcranial Doppler to measure middle cerebral artery blood flow velocity as an indicator of global cerebral blood flow. This approach assumes that the diameter of the intonated vessel remains constant, which has not been confirmed in these experimental settings. Furthermore, transcranial Doppler does not allow assessment of the changes in regional cerebral blood flow, which would require advanced tomographic techniques, such as computed tomography or magnetic resonance imaging. However, given that we aimed to study critically ill patients early in the course of their illness when they are less likely to tolerate transport to a medical imaging department, transcranial Doppler offered a safer alternative that allowed us to monitor changes in global cerebral blood flow with high temporal resolution at the patients' bedside.

Given the exploratory nature of our study and its focus on the feasibility and safety outcomes of passive cycling intervention, we did not collect any long-term clinical outcomes or patient prognosis data. Future properly powered studies in critically ill patients should explore the link between acute changes in hemodynamics and organ perfusion reported in this study and long-term patient clinical and functional outcomes.

## Conclusion

In septic patients, graded passive cycling is associated with dose-dependent decreases in cerebral blood flow, increases in total peripheral resistance, and variable improvement or worsening of left ventricular function. The magnitude and cadence threshold of these responses vary between patients. Future studies should establish whether these changes are associated with clinical outcomes, including cognitive impairment, vasopressor use, and functional outcomes.

## Data Availability Statement

The raw data supporting the conclusions of this article will be made available by the authors, without undue reservation.

## Ethics Statement

The studies involving human participants were reviewed and approved by University of Western Ontario Health Sciences Research Ethics Board. The patients/participants provided their written informed consent to participate in this study.

## Author Contributions

MS, JC, CM, IB, and CWM conceived and designed the research protocol. JC collected and analyzed the data and wrote the first draft of the manuscript. All authors provided input on data analysis and interpretations and participated in multiple revisions of the manuscript.

## Conflict of Interest

The authors declare that the research was conducted in the absence of any commercial or financial relationships that could be construed as a potential conflict of interest.
